# Thoracoscopic Lobectomy Versus Sublobar Resection for pStage I Geriatric Non-Small Cell Lung Cancer

**DOI:** 10.3389/fonc.2021.777590

**Published:** 2022-01-24

**Authors:** Young-Jen Lin, Xu-Heng Chiang, Tzu-Pin Lu, Min-Shu Hsieh, Mong-Wei Lin, Hsao-Hsun Hsu, Jin-Shing Chen

**Affiliations:** ^1^ Department of Surgery, National Taiwan University Hospital, and National Taiwan University College of Medicine, Taipei, Taiwan; ^2^ Institute of Epidemiology and Preventive Medicine, College of Public Health, National Taiwan University, Taipei, Taiwan; ^3^ Department of Pathology, National Taiwan University Hospital, and National Taiwan University College of Medicine, Taipei, Taiwan

**Keywords:** early-stage non-small cell lung cancer, thoracoscopic, lobectomy resection, sublobar resection, overall survival, disease-free survival

## Abstract

**Objectives:**

The choice of resection method for geriatric patients with early-stage non-small cell lung cancer (NSCLC) remains contentious. This study aimed to evaluate survival and perioperative outcomes after thoracoscopic lobectomy resection (LR) or sublobar resection (SR) in patients aged ≥75 years with pathologic stage (pStage) I NSCLC.

**Materials and Methods:**

We retrospectively examined 258 consecutive patients aged ≥75 years with pStage I NSCLC who underwent thoracoscopic tumor resection at our institute from 2011 to 2018. Propensity score matching (PSM) analysis identified 60 patients in each group for comparison of survival-related parameters, including disease-free survival (DFS), lung cancer-specific overall survival (OS), and non-lung cancer-specific OS, using the Kaplan-Meier analysis.

**Results:**

LR and SR were performed in 84 (32.6%) and 174 (67.4%) patients aged ≥75 years, respectively. The LR group had younger patients, better performance status, larger tumor sizes, and deeper tumor location than the SR group. Multivariate studies showed that the resection method was not a prognostic factor for OS. The two PSM-matched groups were not significantly different with respect to lung cancer-specific OS (p = 0.116), non-lung cancer-specific OS (p = 0.408), and DFS (p = 0.597). SR helped achieve better perioperative outcomes than LR, including fewer postoperative complications (10.0% vs. 28.3%, p = 0.011), shorter operative times (p < 0.001), decreased blood loss (p = 0.026), and shorter chest tube duration (p = 0.010) and hospital stays (p = 0.035).

**Conclusions:**

Thoracoscopic SR may provide similar oncological outcomes to LR, but may be a safer and more feasible surgical method for geriatric patients with pStage I NSCLC.

## Introduction

Lung cancer is the leading cause of cancer death (1). The median age at diagnosis of non-small cell lung cancer (NSCLC) is 71 years in the Western world, with 36.3% of cases occurring among individuals aged ≥75 years (1). The detection rate of early-stage cancers in the elderly population is expected to substantially increase with the widespread use of computed tomography (CT) screening (2, 3). The management of early NSCLC in older patients presents a major challenge.

Lobectomy resection (LR) is the standard curative treatment for early-stage NSCLC (4, 5). However, sublobar resection (SR) is recommended for patients with compromised cardiopulmonary function. Recently, several retrospective studies have shown that SR has comparable oncological outcomes, better lung function preservation, and lower perioperative morbidities when compared with LR (6, 7). Thus, SR represents a feasible surgical method for geriatric patients with early NSCLCs (8–14). Despite the high incidence of lung cancer in the elderly population, the preferred resection method (i.e., LR or SR) in patients with early-stage NSCLC is still controversial (4). Therefore, re-evaluation of surgical treatment strategies in these patients is required. No previous study has compared thoracoscopic LR with SR in geriatric patients with early NSCLCs.

This retrospective study aimed to compare perioperative outcomes and survival of patients who underwent thoracoscopic SR or LR, and to identify prognostic factors in geriatric patients aged ≥75 years with surgically resected pStage I NSCLC.

## Material and Methods

### Study Population

**Figure 1:**
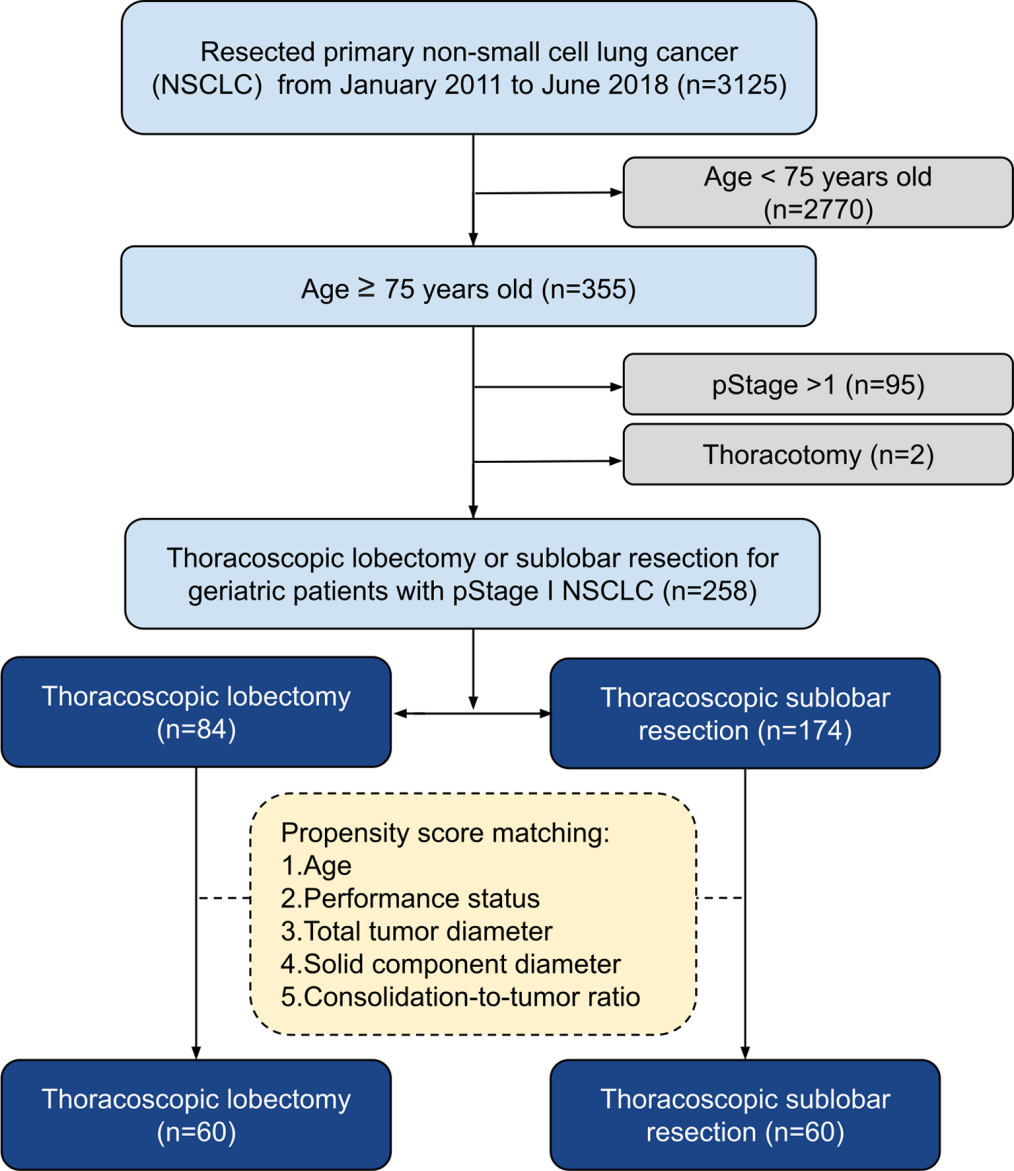
Algorithm for patient selection. NSCLC, Non-small cell lung cancer.

We retrospectively examined 3,125 consecutive patients with NSCLC who underwent surgical treatments by a single surgical team at the National Taiwan University Hospital from January 2011 to June 2018. The inclusion criteria were pStage I NSCLC, age ≥75 years, and patients undergoing thoracoscopic LR or SR. In total, 258 patients were included in the study (**Figure 1**). Our hospital research ethics committee approved this retrospective study (202005006RINC) and waived the requirement for informed consent.

Preoperative clinical data was obtained from a prospectively collected database at our institute. Preoperative comorbidities were evaluated according to the Charlson Comorbidity Index (15). All preoperative studies were performed at our institute within two months before the operation, including pulmonary function test, echocardiography, test for preoperative serum carcinoembryonic antigen (CEA) levels, chest radiography, contrast-enhanced chest/abdomen CT, brain CT or magnetic resonance imaging (MRI), bone scanning, and positron emission tomography (PET). Tumor depth was defined as the shortest distance from the tumor to the pleura. The consolidation-to-tumor ratio (C/T ratio) was the ratio of the maximum diameter of consolidation to the maximum tumor diameter, measured by using CT according to the Japan Clinical Oncology Group 0201 definition (16). All imaging studies were independently reviewed by two thoracic surgeons in our surgical team using standard pictures with a commercially available viewer (IMPAX 5.2; Agfa HealthCare N.V., Mortsel, Belgium).

At our institute, SR was indicated if the tumor diameter was ≤2 cm and the C/T ratio was ≤50% in patients with NSCLC without lymph node or distant metastases. SR was considered if the resection margin was at >2 cm from than the tumor diameter. Otherwise, LR was performed to achieve the appropriate resection margin, which was defined based on gross measurements during surgery. Each surgeon made the decision for which surgical method (i.e., LR or SR) to use, which was then approved during a weekly multidisciplinary lung cancer meeting attended by surgeons, radiologists, pathologists, and oncologists before the surgery. After the SR was completed, mediastinal lymph nodes were sampled, depending on the location of the lung nodule, for lung cancer staging. The severity of postoperative complications was graded according to the Clavien-Dindo classification, which classifies postoperative complications as requiring pharmacological treatment (Grade II), surgical or radiological intervention (Grade III), or life-threatening complication (Grade IV) (17).

The histological diagnosis and pathological features were retrospectively collected from pathological analysis documents at the National Taiwan University Hospital. The pathological staging of lung tumors was determined according to the 8th Edition of the American Joint Committee on Cancer TNM staging system (18). The histopathology pattern was classified according to the 2015 World Health Organization criteria (19). Tumor spread through air spaces (STAS) was defined as tumor cells within air spaces in the lung parenchyma at a distance of at least one alveolus away from the main tumor (20).

### Patient Follow-Up and Outcome Measurement

Patients were monitored postoperatively in the outpatient clinic with physical examinations, serum CEA measurements, and chest CT examinations every 6 months for the first 2 years and every 6–12 months thereafter. Lung cancer recurrence was initially assessed using CT, PET, and MRI. It was further confirmed using CT-guided, endobronchial ultrasound-guided, and transthoracic echogram-guided biopsies or pleural effusion tapping.

For patients who underwent sublobar resection with an involved margin, the sequential treatments are completion lobectomy or radiotherapy within eight weeks. Adjuvant cisplatin-based chemotherapy is indicated for patients with nodal metastasis. Radiation therapy dose of 45-60 Gy as the highest cumulative radiation dose is indicated for lymph node metastases with extranodal extension.

The classification of the cause of death were lung cancer specific and non-lung cancer specific. Lung cancer-specific mortality was defined as death owing to recurrent disease associated with resected NSCLCs. Non-lung cancer-specific mortality was defined as death owing to specific causes other than NSCLCs. Death as a result of other malignancies was also regarded as non-lung cancer-specific death.

### Propensity Score Matching (PSM)

A propensity score matching (PSM) analysis was adopted to select comparable cases in two groups for further analyses and to remove the potential impact of confounding factors. Briefly, the propensity score was calculated using a multivariate logistic regression model. Age, performance status, total tumor size, solid tumor size, and C/T ratio were included as covariates. Patients undergoing LR were matched to those undergoing SR in a 1:1 ratio, with a standardized mean difference (SMD) of <0.2 of the logit propensity score (21). The PSM algorithm was executed with PSM for the Statistical Package for the Social Sciences (SPSS) (add-on for SPSS, version 3.04) and the underlying R packages (version 3.3.0).

### Statistical Analyses

Descriptive statistics are reported as mean ± standard deviation for continuous data and as percentages (%) for categorical data. Before PSM, chi-squared tests were performed for categorical variables, and Student’s t-test was performed for continuous variables. These values were also estimated after PSM using chi-squared tests for categorical variables and paired t-tests for continuous data. Overall survival (OS) and disease-free survival (DFS) rates were calculated using the Kaplan-Meier method and analyzed using log-rank tests. Multivariate analyses were performed using the Cox regression model after adjusting for significant confounding factors in the univariate model. Statistical analyses were performed using SPSS version 25 (IBM, Armonk, New York, USA). All tests were two sided, and p-values <0.05 were considered significant.

## Results

### Patient Demographics and Clinicopathological Features

The study cohort included 84 (32.6%) patients who underwent LR and 174 (67.4%) patients (including 128 wedge resections and 46 segmentectomies) who underwent SR. The mean follow-up period was 39.8 ± 24.6 months. The mean age of all 258 patients was 78.6 ± 3.3 years (range: 75-90). The majority of patients were female (57.0%) and non-smoker (79.5%). The comorbidity index of the study group was 1.5 ± 1.4. The details of patient demographics and clinical characteristics of the study cohort are listed in **Supplementary Table 1**.

Before PSM, patients in the LR group were younger (p = 0.013) and had a better Eastern Cooperative Oncology Group performance status (p = 0.007) than those in the SR group. The total tumor diameter, solid component diameter, and tumor depth were significantly larger in the LR group than in the SR group (**Supplementary Table 1**). The pathological features showed that the pathological tumor diameter and pStage were significantly different between the two groups. The LR group had a larger pathological tumor diameter (p < 0.001) and a higher presence of IB pStage (p < 0.001) than the SR group (**Supplementary Table 2**). There was no significant difference in positive STAS between lobectomy and sublobar resection group in this study cohort (42.1% vs. 31.5%, p=0.577).

PSM was used to identify 60 well-balanced patients in each group for a survival comparison. After PSM, only the tumor depth was greater in the LR group than in the SR group (p = 0.001). No other differences in demographic, clinical, histopathologic features, and pStage were observed between the two groups (**Tables 1**, **2**). The distribution of the propensity scores and SMDs for both groups before and after PSM is shown in **Supplementary Figure 1**.

**Table 1:** Demographic and clinical features after propensity score matching.

	All	Lobectomy	Sublobar	P-value
	(n = 120)	(n = 60)	(n = 60)	
Age, years	78.1 ± 2.9	78.2 ± 2.9	78.1 ± 3.0	.74
Male	49 (40.8)	29 (48.3)	20 (33.3)	.10
BMI	24.6 ± 3.2	24.5 ± 2.8	24.6 ± 3.5	.92
Smoking status				.82
Smoker	25 (20.8)	12 (20.0)	13 (21.7)	
Non-smoker	95 (79.2)	48 (80.0)	47 (78.3)	
Lung cancer family history				.14
Yes	13 (10.8)	9 (15.0)	4 (6.7)	
No	107 (89.2)	51 (85.0)	56 (93.3)	
ECOG				.44
0	78 (65.0)	37 (61.7)	41 (68.3)	
≥1	42 (35.0)	23 (38.3)	19 (31.7)	
Comorbidity index (CCI)	1.5 ± 1.4	1.4 ± 1.3	1.6 ± 1.5	.27
PFT				
FVC, %	109.5 ± 19.9	109.9 ± 19.1	109.0 ± 20.7	.80
FEV1, %	117.3 ± 27.3	116.6 ± 28.7	117.9 ± 26.1	.80
CEA^a^				.57
≥5 ng/mL	16 (13.7)	7 (11.9)	9 (15.5)	
<5 ng/mL	101 (86.3)	52 (88.1)	49 (84.5)	
Total tumor diameter, cm				.39
0–1	6 (5.0)	1 (1.7)	5 (8.3)	
1–2	34 (28.3)	17 (28.3)	17 (28.3)	
2–3	53 (44.2)	27 (45.0)	26 (43.3)	
≥3	27 (22.5)	15 (25.0)	12 (20.0)	
Solid component diameter, cm				.11
0–1	23 (19.2)	7 (11.7)	16 (26.7)	
1–2	52 (43.3)	29 (48.3)	23 (38.3)	
≥2	45 (37.5)	24 (40.0)	21 (35.0)	
C/T ratio (%)	44.6 ± 43.2	47.7 ± 43.7	41.4 ± 42.8	.33
0–25%	56 (46.7)	26 (43.3)	30 (50.0)	
25–50%	8 (6.7)	3 (5.0)	5 (8.3)	
≥50%	56 (46.7)	31 (51.7)	25 (41.7)	
Tumor depth, cm	0.9 ± 0.8	1.2 ± 0.9	0.6 ± 0.6	.001

Data is presented as mean ± standard deviation or number (%).

BMI, body mass index; CCI, Charlson Comorbidity Index; CEA, carcinoembryonic antigen; C/T ratio, consolidation-to-tumor ratio; ECOG, Eastern Cooperative Oncology Group performance status; FEV1, forced expiratory volume in 1 second; FVC, forced vital capacity; PFT, pulmonary function test. ^a^3 patients lack the preoperative serum CEA level data.

**Table 2:** Pathological features after propensity score matching.

	All	Lobectomy	Sublobar	P-value
	(n = 120)	(n = 60)	(n = 60)	
Differentiation^a^				.84
Well	21 (17.9)	11 (18.6)	10 (17.2)	
Moderate poor	96 (82.1)	48 (81.4)	48 (82.8)	
VPI	24 (20.0)	12 (50.0)	12 (50.0)	>.99
LVI	17 (14.2)	9 (15.0)	8 (13.3.)	.79
Pathological tumor diameter, cm				.19
0–1	4 (3.3)	2 (3.3)	2 (3.3)	
1–2	34 (28.3)	13 (21.7)	21 (35.0)	
2–3	46 (38.3)	22 (36.7)	24 (40.0)	
≥3	36 (30.0)	23 (38.3)	13 (21.7)	
Histology				.64
Adenocarcinoma	106 (88.3)	51 (85.0)	55 (91.7)	
SqCC	10 (8.3)	6 (10.0)	4 (6.7)	
Adenosquamous	2 (1.7)	1 (1.7)	1 (1.7)	
Pleomorphic	1 (0.8)	1 (1.7)	0	
Carcinoid	1 (0.8)	1 (1.7)	0	
Pathological stage				.16
IA	73 (60.8)	33 (55.0)	40 (66.7)	
IA1	8 (6.7)	3 (5.0)	5 (8.3)	
IA2	33 (27.5)	13 (21.7)	20 (33.3)	
IA3	47 (39.2)	23 (38.3)	24 (40.0)	
IB	47 (39.2)	27 (45.0)	20 (33.3)	
Resection margin involvement	5 (4.2)	1 (1.7)	4 (6.7)	.17

Data is presented as mean ± standard deviation or number (%).

LVI, lymphovascular invasion; SqCC, squamous cell carcinoma; VPI, visceral pleural. ^a^patients lack the pathology differentiation data.

### Operative and Perioperative Results

There was no 30-day mortality in the study cohort, and only one patient in the LR group underwent conversion to thoracotomy. After matching, SR was associated with better perioperative outcomes, including lower blood loss and shorter operative times, hospital stays, and chest tube duration (**Table 3** and **Supplementary Table 3**). Postoperative complications were less common in the SR group than in the LR group (10.0% vs. 28.3%, p = 0.011). Severe postoperative complications with a Clavien-Dindo classification (17) greater than 3a were also less common in the SR group than in the LR group (10.0% vs. 25.0%, p = 0.031). The details of postoperative complications are listed in **Supplementary Table 4**.

**Table 3:** Perioperative outcomes after propensity score matching.

	All	Lobectomy	Sublobar	P-value
	(n = 120)	(n = 60)	(n = 60)	
VATS approach	120 (100.0)	60 (100.0)	60 (100.0)	>.99
Operative time, min	118.5 ± 45.9	140 ± 43.3	97.8 ± 38.4	<.001
Operative bleeding, mL	19.5 ± 58.0	31.3 ± 74.2	7.7 ± 31.6	.03
Postoperative hospital stay, days	6.6 ± 7.9	8.0 ± 8.0	5.3 ± 5.1	.04
Postoperative ICU stay, days	0.7 ± 1.4	0.6 ± 0.6	0.8 ± 1.9	.49
Dissected lymph nodes				
Total number	9.3 ± 7.4	11.5 ± 7.5	7.1 ± 6.6	<.001
Total station	3.5 ± 1.5	3.9 ± 1.3	3.1 ± 1.6	.003
Chest tube				
Chest tube duration, days	3.1 ± 3.7	4.0 ± 4.7	2.3 ± 1.8	.01
Chest tube ≥3 days	46 (38.3)	33 (55.0)	13 (21.7)	<.001
Chest tube >5 days	13 (10.8)	10 (16.3)	3 (5.0)	.04
Postoperative complications				
All complications	23 (19.2)	17 (28.3)	6 (10.0)	.01
Grade 3a or greater	21 (17.5)	15 (25.0)	6 (10.0)	.03
Grade 3b or greater	4 (3.3)	3 (5.0)	1 (1.7)	.31
Conversion to thoracotomy	0	0	0	>.99
30-day mortality	0	0	0	>.99

Data is presented as mean ± standard deviation or number (%).

ICU, intensive care unit; NA, not available; TIA, transient ischemic attack; VATS, video-assisted thoracoscopic surgery.

### Comparison of Clinical Outcomes Before and After PSM Analysis

Our results showed favorable clinical outcomes for both thoracoscopic LR and SR in geriatric patients with pStage I NSCLC. The Kaplan-Meier OS analysis showed no difference between the LR and SR groups with respect to lung cancer-specific OS (5-year lung cancer-specific OS: 100% vs. 98.2%, p = 0.081), non-lung cancer-specific OS (5-year non-lung cancer-specific OS: 83.9% vs. 90.7%, p = 0.853), and DFS (5-year DFS: 78.8% vs. 82.4%; *p* = 0.643). Within the follow-up period, 33 patients (12.8%) developed recurrence, among whom, 14 underwent LR and 19 underwent SR (**Supplementary Table 5**).

In this study cohort, 11 patients received SR with microscopic resection margin involvement, and only one patient (9.1%, 1/11) received reresection. The rest of the patients received radiotherapy for positive resection margin. In total, 11 patients developed local recurrence, 27.3% (3/11) patients received reresection, and the remaining received adjuvant therapy (tyrosine kinase inhibitor for mutant epidermal growth factor receptor [EGFR], or chemotherapy for wild-type EGFR mutation) plus radiotherapy.

**Figure 2:**
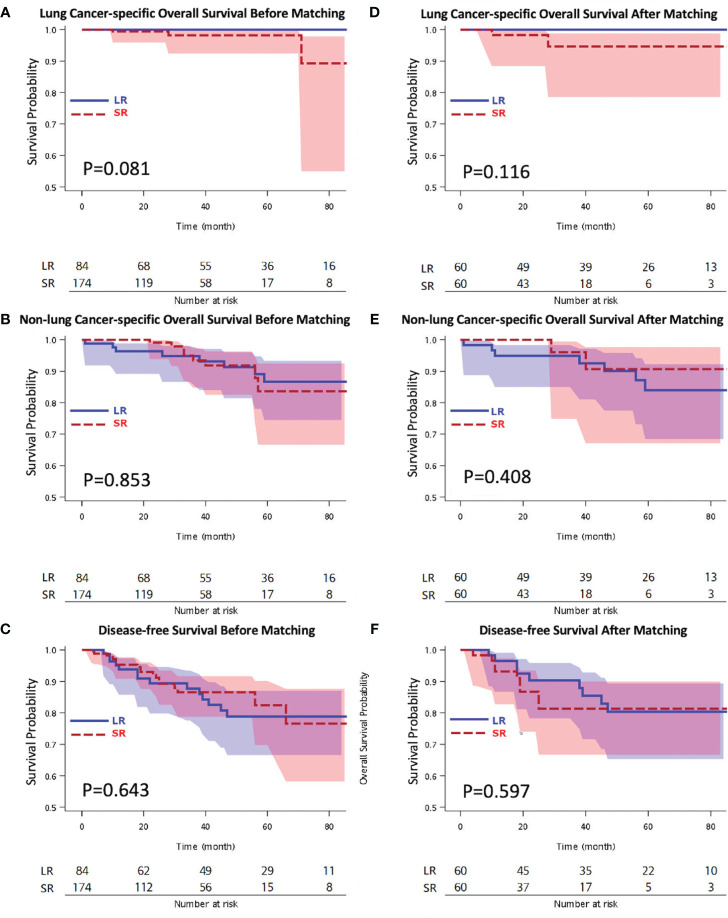
Kaplan-Meier overall survival by **(A)** lung cancer-specific overall survival survival, **(B)** non-lung cancer-specific overall survival, **(C)** disease-free survival analyses before matching, and **(D)** lung cancer-specific overall survival, **(E)** non-lung cancer-specific overall survival, **(F)** disease-free survival analyses after matching. LR, lobectomy; SR, sublobar resection.

After matching, the Kaplan-Meier OS analysis showed no difference between the two groups with respect to both lung cancer-specific OS (LR vs. SR; 5-year lung cancer-specific OS: 100% vs. 94.6%, p = 0.116) and non-lung cancer-specific OS (LR vs. SR; 5-year non-lung cancer-specific OS: 83.9% vs. 90.7%, p = 0.408) (**Figure 2**). The Kaplan-Meier DFS analysis also showed no significant difference between the LR and SR groups (5-year DFS: 80.3% vs. 81.3%; p = 0.597) (**Figure 2**). The causes of mortality stratified according to the lung cancer specific and non-lung cancer specific variables are shown in **Supplementary Table 6**. There was no significant difference in the cause of mortality between the LR and SR groups (p = 0.356).

### Correlation Between Clinicopathological Features, Surgical Methods, and Clinical Outcomes

The results of the univariate analysis, which examined the correlation between clinicopathological features and OS, are listed in **Supplementary Tables 7–9**. We evaluated both lung cancer-specific and non-lung cancer-specific survival. Because of the limited number of events, no multivariate analysis was performed for lung cancer-specific OS. The multivariate analysis results are listed in **Supplementary Table 9**. Female sex (p = 0.049) was the only independent factor of non-cancer-specific death and was associated with increased survival. The resection method was not a prognostic factor for lung cancer-specific and non-lung cancer-specific OS.

## Discussion

SR, including wedge resection and segmentectomy, is often offered to patients with comorbid conditions, such as poor cardiopulmonary function or physiological impairments, who would not be able to tolerate standard LR. The recommendation was established by The Lung Cancer Study Group trial published in 1995, which is the only randomized controlled trial that compared LR and SR for stage I NSCLC (5) and suggested that local recurrence is significantly more frequent with SR than with LR. To this day, LR remains the “gold standard” for the curative treatment of lung cancer in patients with adequate cardiorespiratory reserve (4). An increasing number of studies suggest that SR may achieve equivalent oncological outcomes as LR for early-stage NSCLC (6, 7). However, few studies have compared SR to LR for the surgical treatment of geriatric patients with NSCLC (8–14, 22, 23). Besides, no previous study has compared thoracoscopic LR with SR in geriatric patients with early-stage NSCLCs. In this study we focused on geriatric patients aged ≥75 years with pStage I NSCLC. The PSM analysis showed that thoracoscopic SR achieved comparable oncological outcomes to thoracoscopic LR with respect to DFS, lung cancer-specific OS, and non-lung cancer-specific OS in patients with pStage I NSCLC. Thoracoscopic SR resulted in better perioperative outcomes than thoracoscopic LR, and multivariate analyses showed that the resection method was not a significant prognostic factor for OS.

SR showed similar oncological outcomes to LR in geriatric patients with early-stage NSCLC (8–14). However, this was contradicted by the inferior survival in the SR group of other studies (22, 23). In 2005, Mery et al. (9) reported that SR with adequate margins might be an alternative for the curative treatment of older patients (aged >71 years) since it did not significantly affect their long-term survival. In 2010, Wisnivesky et al. (10) reported a similar survival rate in patients (aged >65 years) undergoing limited resection or lobectomy for stage IA tumors ≤2 cm, and they suggested that SR may be an effective therapeutic alternative for these patients. However, Shirvani et al. (22) have reported that SR was significantly associated with worse OS than LR in older patients with early-stage NSCLC. Meanwhile, Veluswamy et al. (23) reported that LR achieves results equivalent to that of segmentectomy but not wedge resection for early-stage lung adenocarcinoma. In the past 5 years, four studies showed no significant difference in the clinical outcome between SR and LR, and SR may be an acceptable oncological procedure for geriatric patients with clinical Stage I NSCLC (11–14). The details of these four studies are summarized in **Table 4**. Our study is the first to compare thoracoscopic LR vs. SR in geriatric patients with early-stage NSCLCs and confirm the results of these previous studies.

**Table 4:** Summary of studies discussing the survival correlation of lobectomy vs. sublobar resection in patients with geriatric early-stage lung cancer in recent 5 years.

Published year	Study period	No. of patients		VATS	Age	Stage	Survival difference between LR and SR group	
		LR	SR (Seg/wedge)				OS	DFS
2021 [current study]	2011–2018	84	174 (46/128)	100%	≥75	pStage I NSCLC	No significant difference in both lung cancer-specific and non-lung cancer-specific OS	No significant difference
2019 (11)	2014–2017	136	106 (20/86)	84.3%	≥75	cStage I NSCLC	No significant difference	No significant difference
2018 (12)	2007–2015	106	99 (56/43)	NA	≥75	cStage I NSCLC	No significant difference	No significant difference
2018 (13)	2006–2014	156	76 (50/26)	NA	≥75	cStage I NSCLC	No significant difference	No significant difference
2018 (14)	1998–2015	237	94 (28/66)^a^	43.3%	≥80	pStage IA-IIIA NSCLC	NA (Surgical method was not a prognostic factor.)	NA

LR, lobectomy; NA, not available; NSCLC, non-small cell lung carcinoma; OS, overall survival; SR, sublobar resection; VATS, video-assisted thoracoscopic surgery.

a In this study, five and one patients underwent bilobectomy and pneumonectomy, respectively.

In this study, SR had a better perioperative outcome than LR, including fewer postoperative complications, decreased blood loss, and shorter operative times and hospital stays. These results are comparable with those of previous studies; the limited resection in SR when compared with that in LR was associated with comparable oncological efficacy, reduced surgical risks, and shorter hospital stays (24). Moreover, SR in geriatric patients with stage I NSCLC has been associated with less severe postoperative complications and OS than LR (11–13). The overall postoperative complication rate of 14.3% in this study was considerably lower than that previously reported (53.9%) in geriatric patients (13), which may be because of the minimally invasive thoracoscopy approach used in this study. The comparable oncological outcome suggests that the less invasive thoracoscopic SR could benefit older patients as it preserves lung parenchyma, leading to a faster recovery. However, more studies have to be conducted in the future to better understand these differences.

Among clinical characteristics, multivariate analyses revealed that gender is the only significant prognostic factor. Females were associated with better survival outcomes, consistent with the results of previously reported case series (25–27). Age and surgical methods were not significantly correlated with the clinical outcome in this study, consistent with the finding of a previous study by Sigel et al. (26), who showed that age alone was not correlated with a higher incidence of mortality or morbidity. This study did not have a 30-day hospital mortality, whereas previously reported mortality rates ranged from 1.2% to 10% (27). Possible reasons for the improved non-lung cancer-related and lung cancer-specific OS in this study may be the minimally invasive thoracoscopic surgery and early CT screening that detected small lung tumors. These results suggest that thoracoscopic surgery for geriatric patients with early-stage NSCLC is safe with promising outcomes. The findings support the concept proposed by Sigel et al. (26) that geriatric patients with stage I lung cancer may undergo aggressive surgery.

This study has limitations and biases. The inherent bias associated with retrospective studies could not be avoided, especially regarding the selection of the surgical method by the surgical team. We used PSM and multivariate analyses to reduce the selection bias. The sublobar resection included segmentectomy and wedge resection. The choice between wedge resection and segmentectomy as a sublobar resection method for geriatric patients may need further evaluation by multi-institutional studies. The study cohort was exclusively Asian, with a high percentage of patients having adenocarcinoma (88%); thus, our findings should carefully be extrapolated to other NSCLC populations. Despite these limitations, we provided detailed, comprehensive, and accurate data obtained from a prospectively collected database at our institute. For clinicians who manage geriatric patients with stage I NSCLC, the study provides clinically relevant information.

In conclusion, our results showed that thoracoscopic SR might provide similar oncological outcomes to LR and help obtain better perioperative outcomes than lobectomies. Thoracoscopic SR may be a safe and feasible surgical method for geriatric patients with pStage I NSCLC and can be the treatment of choice for these patients. The results of our study should be further validated in ongoing prospective, multi-institutional studies in the near future (28–30).

## Data Availability Statement

The original contributions presented in the study are included in the article/**Supplementary Material**. Further inquiries can be directed to the corresponding author.

## Ethics Statement

The studies involving human participants were reviewed and approved by National Taiwan University Hospital research ethics committee (202005006RINC). The patients/participants provided their written informed consent to participate in this study. Written informed consent was obtained from the individual(s) for the publication of any potentially identifiable images or data included in this article.

## Author Contributions

M-WL, H-HH, and J-SC contributed to conception and design of the study. Y-JL, X-HC, T-PL, M-SH, and M-WL organized the database. Y-JL, X-HC, T-PL, and M-WL performed the statistical analysis. Y-JL, X-HC, M-SH, and M-WL wrote the first draft of the manuscript. All authors contributed to manuscript revision, read, and approved the submitted version.

## Funding

The authors declare that this study received funding from the National Taiwan University Hospital Taipei, Taiwan (NTUH110-S5037) and the Ministry of Science and Technology, Taiwan (MOST110-2314-B-002-271) and Taiwan Lung Foundation. The funder had the following involvement with the study: English language editing and article processing fee. The funder was not involved in the study design, collection, analysis, interpretation of data, the writing of this article, or the decision to submit it for publication.

## Conflict of Interest

The authors declare that the research was conducted in the absence of any commercial or financial relationships that could be construed as a potential conflict of interest.

## Publisher’s Note

All claims expressed in this article are solely those of the authors and do not necessarily represent those of their affiliated organizations, or those of the publisher, the editors and the reviewers. Any product that may be evaluated in this article, or claim that may be made by its manufacturer, is not guaranteed or endorsed by the publisher.

## References

[1] SEER. Cancer Stat Facts 2021: Lung and Bronchus Cancer . Available at: http://seer.cancer.gov/statfacts/html/lungb.html (Accessed April 24, 2021).

[2] de Koning HJ van der Aalst CM de Jong PA Scholten ET Nackaerts K Heuvelmans MA. Reduced Lung-Cancer Mortality With Volume CT Screening in a Randomized Trial. N Engl J Med (2020) 382:503–13. doi: 10.1056/NEJMoa1911793 31995683

[3] Lin MW Tseng YH Lee YF Hsieh MS Ko WC Chen JY. Computed Tomography-Guided Patent Blue Vital Dye Localization of Pulmonary Nodules in Uniportal Thoracoscopy. J Thorac Cardiovasc Surg (2016) 152:535–44. doi: 10.1016/j.jtcvs.2016.04.052 27189890

[4] Howington JA Blum MG Chang AC Balekian AA Murthy SC. Treatment of Stage I and II Non-Small Cell Lung Cancer: Diagnosis and Management of Lung Cancer, 3rd Ed: American College of Chest Physicians Evidence-Based Clinical Practice Guidelines. Chest (2013) 143:e278S–313S. doi: 10.1378/chest.12-2359 23649443

[5] Ginsberg RJ Rubinstein LV. Lung Cancer Study Group, Randomized Trial of Lobectomy Versus Limited Resection for T1N0 Non-Small Cell Lung Cancer. Ann Thorac Surg (1995) 60:615–22. doi: 10.1016/0003-4975(95)00537-u 7677489

[6] Altorki NK Yip R Hanaoka T Bauer T Aye R Kohman L. Sublobar Resection is Equivalent to Lobectomy for Clinical Stage 1A Lung Cancer in Solid Nodules. J Thorac Cardiovasc Surg (2014) 147:754–64. doi: 10.1016/j.jtcvs.2013.09.065 24280722

[7] Chiang XH Hsu HH Hsieh MS Chang CH Tsai TM Liao HC. Propensity-Matched Analysis Comparing Survival After Sublobar Resection and Lobectomy for Ct1n0 Lung Adenocarcinoma. Ann Surg Oncol (2020) 27:703–15. doi: 10.1245/s10434-019-07974-9 31646453

[8] Landreneau RJ Normolle DP Christie NA Awais O Wizorek JJ Abbas G. Recurrence and Survival Outcomes After Anatomic Segmentectomy Versus Lobectomy for Clinical Stage I Non-Small-Cell Lung Cancer: A Propensity-Matched Analysis. J Clin Oncol (2014) 32:2449–55. doi: 10.1200/JCO.2013.50.8762 PMC4121502 24982447

[9] Mery CM Pappas AN Bueno R Colson YL Linden P Sugarbaker DJ. Similar Long-Term Survival of Elderly Patients With Non-Small Cell Lung Cancer Treated With Lobectomy or Wedge Resection Within the Surveillance, Epidemiology and End-Results Database. Chest (2005) 128:237–45. doi: 10.1378/chest.128.1.237 16002941

[10] Wisnivesky JP Henschke CI Swanson S Yankelevitz DF Zulueta J Marcus S. Limited Resection for the Treatment of Patients With Stage IA Lung Cancer. Ann Surg (2010) 251:550–4. doi: 10.1097/SLA.0b013e3181c0e5f3 20160639

[11] Zhang Z Feng H Zhao H Hu J Liu L Liu Y. Sublobar Resection is Associated With Better Perioperative Outcomes in Elderly Patients With Clinical Stage I Non-Small Cell Lung Cancer: A Multicenter Retrospective Cohort Study. J Thorac Dis (2019) 11:1838–48. doi: 10.21037/jtd.2019.05.20 PMC6588758 31285876

[12] Sutani Y Tsubokawa N Ito M Misumi K Hanaki H Miyata Y. Postoperative Complications and Prognosis After Lobar Resection Versus Sublobar Resection in Elderly Patients With Clinical Stage I Non-Small-Cell Lung Cancer. Eur J Cardiothorac Surg (2018) 53:366–71. doi: 10.1093/ejcts/ezx296 28958068

[13] Yutaka Y Sonobe M Kawaguchi A Hamaji M Nakajima D Ohsumi A. Prognostic Impact of Preoperative Comorbidities in Geriatric Patients With Early-Stage Lung Cancer: Significance of Sublobar Resection as a Compromise Procedure. Lung Cancer (2018) 125:192–7. doi: 10.1016/j.lungcan.2018.09.023 30429019

[14] Hino H Karasaki T Yoshida Y Fukami T Sano A Tanaka M. Risk Factors for Postoperative Complications and Long-Term Survival in Lung Cancer Patients Older Than 80 Years. Eur J Cardiothorac Surg (2018) 53:980–6. doi: 10.1093/ejcts/ezx437 29272371

[15] Charlson ME Pompei P Ales KL MacKenzie CR. A New Method of Classifying Prognostic Comorbidity in Longitudinal Studies: Development and Validation. J Chronic Dis (1987) 40:373–83. doi: 10.1016/0021-9681(87)90171-8 3558716

[16] Suzuki K Koike T Asakawa T Kusumoto M Asamura H Nagai K. A Prospective Radiological Study of Thin-Section Computed Tomography to Predict Pathological Noninvasiveness in Peripheral Clinical IA Lung Cancer (Japan Clinical Oncology Group 0201). J Thorac Oncol (2011) 6:751–6. doi: 10.1097/JTO.0b013e31821038ab 21325976

[17] Clavien PA Barkun J De Oliveira ML Vauthey JN Dindo D Schulick RD. The Clavien-Dindo Classification of Surgical Complications: Five-Year Experience. Ann Surg (2009) 250:187–96. doi: 10.1097/SLA.0b013e3181b13ca2 19638912

[18] Detterbeck FC Boffa DJ Kim AW Tanoue LT. The Eighth Edition Lung Cancer Stage Classification. Chest (2017) 151:193–203. doi: 10.1016/j.chest.2016.10.010 27780786

[19] Travis WD Brambilla E Nicholson AG Yatabe Y Austin JH Beasley MB. The 2015 World Health Organization Classification of Lung Tumors: Impact of Genetic, Clinical and Radiologic Advances Since the 2004 Classification. J Thorac Oncol (2015) 10:1243–60. doi: 10.1097/JTO.0000000000000630 26291008

[20] Hu SY Hsieh MS Hsu HH Tsai T-M Chiang X-H Tsou K-C. Correlation of Tumor Spread Through Air Spaces and Clinicopathological Characteristics in Surgically Resected Lung Adenocarcinomas. Lung Cancer (2018) 126:189–93. doi: 10.1016/j.lungcan.2018.11.003 30527186

[21] Grose E Wilson S Barkun J Bertens K Martel G Balaa F. Use of Propensity Score Methodology in Contemporary High-Impact Surgical Literature. J Am Coll Surg (2020) 230:101–12. doi: 10.1016/j.jamcollsurg.2019.10.003 31672675

[22] Shirvani SM Jiang J Chang JY Welsh J Likhacheva A Buchholz TA. Lobectomy, Sublobar Resection, and Stereotactic Ablative Radiotherapy for Early-Stage Non-Small Cell Lung Cancers in the Elderly. JAMA Surg (2014) 149:1244. doi: 10.1001/jamasurg.2014.556 25321323 PMC4401470

[23] Veluswamy RR Ezer N Mhango G Goodman E Bonomi M Neugut AI. Limited Resection Versus Lobectomy for Older Patients With Early-Stage Lung Cancer: Impact of Histology. J Clin Oncol (2015) 33:3447–53. doi: 10.1200/JCO.2014.60.6624 PMC4606062 26240229

[24] Kilic A Schuchert MJ Pettiford BL Pennathur A Landreneau JR Landreneau JP. Anatomic Segmentectomy for Stage I Nn-Small Cell Lung Cancer in the Elderly. Ann Thorac Surg (2009) 87:1662–8. doi: 10.1016/j.athoracsur.2009.02.097 19463574

[25] Sagerup CM Småstuen M Johannesen TB Helland Å Brustugun OT. Sex-Specific Trends in Lung Cancer Incidence and Survival: A Population Study of 40,118 Cases. Thorax (2011) 66:301–7. doi: 10.1136/thx.2010.151621 21199818

[26] Sigel K Bonomi M Packer S Wisnivesky J. Effect of Age on Survival of Clinical Stage I Non-Small-Cell Lung Cancer. Ann Surg Oncol (2009) 16:1912–7. doi: 10.1245/s10434-009-0475-8 19408051

[27] Kates M Perez X Gribetz J Swanson SJ McGinn T Wisnivesky JP. Validation of a Model to Predict Perioperative Mortality From Lung Cancer Resection in the Elderly. Am J Respir Crit Care Med (2009) 179:390–5. doi: 10.1164/rccm.200808-1342OC 19029001

[28] Altorki NK Wang X Wigle D Gu L Darling G Ashrafi AS. Perioperative Mortality and Morbidity After Sublobar Versus Lobar Resection for Early-Stage Non-Small-Cell Lung Cancer: *Post-Hoc* Analysis of an International, Randomised, Phase 3 Trial (CALGB/Alliance 140503). Lancet Respir Med (2018) 6:915–24. doi: 10.1016/S2213-2600(18)30411-9 PMC6396275 30442588

[29] Nakamura K Saji H Nakajima R Okada M Asamura H Shibata T. A Phase III Randomized Trial of Lobectomy Versus Limited Resection for Small-Sized Peripheral Non-Small Cell Lung Cancer (JCOG0802/WJOG4607L). Jpn J Clin Oncol (2010) 40:271–4. doi: 10.1093/jjco/hyp156 19933688

[30] Yang F Sui X Chen X Zhang L Wang X Wang S. Sublobar Resection Versus Lobectomy in Surgical Treatment of Elderly Patients With Early-Stage Non-Small Cell Lung Cancer (STEPS): Study Protocol for a Randomized Controlled Trial. Trials (2016) 17:191. doi: 10.1186/s13063-016-1312-6 27053091 PMC4823889

