# Supplementary material for: Thoracoscopic Lobectomy Versus Sublobar Resection for pStage I Geriatric Non-Small Cell Lung Cancer
Source: Front Oncol. 2022 Jan 24;11:777590. doi: 10.3389/fonc.2021.777590 (PMC8818756; doi:10.3389/fonc.2021.777590)
Supplement: Supplementary file 1 [file Image_1.pdf]

Supplemental Figure 1.

Summary characteristics of the propensity score matching analysis. A and B: distributions of the propensity scores in both groups before and after matching, respectively; C: distribution of standardized differences in the matched variables before and after matching; D: after matching, the two groups achieved minimal standardized differences (0.2) in the matched variables.

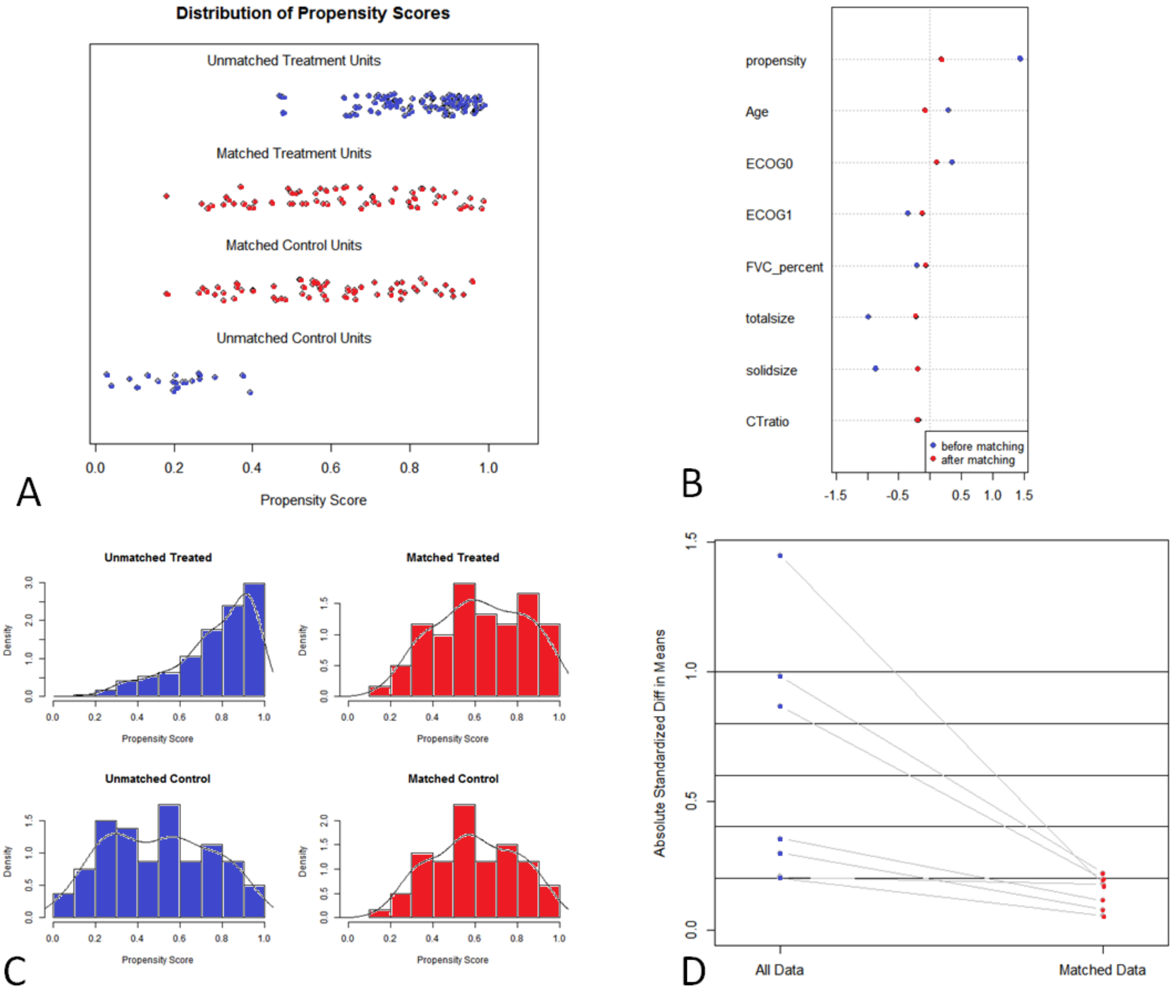

The detail of standardized mean differences values before and after matching were listed below.

	SMD before matching	SMD after matching
Age, yr	0.330	-0.034
ECOG =0	0.361	0.139
ECOG >1	-0.361	-0.139
FVC, %	-0.202	-0.045
Total tumor diameter, cm	-1.178	-0.123
Solid component diameter, cm	-0.868	-0.126
C/T ratio	-0.223	-0.146

Abbreviations: C/T ratio, consolidation-to-tumor ratio; ECOG, Eastern Cooperative Oncology Group performance status; FVC, forced vital capacity; SMD, standardized mean differences.
