# Supplementary material for: Thoracoscopic Lobectomy Versus Sublobar Resection for pStage I Geriatric Non-Small Cell Lung Cancer
Source: Front Oncol. 2022 Jan 24;11:777590. doi: 10.3389/fonc.2021.777590 (PMC8818756; doi:10.3389/fonc.2021.777590)
Supplement: Supplementary file 2 [file Table_1.docx]

**Supplementary Table 1.** Demographic and clinical features before propensity score matching

|  | Total  (n=258) | Lobectomy (n=84) | Sublobar resection  (n=174) | | P-value |
| --- | --- | --- | --- | --- | --- |
|  |  |  | Segmentectomy  (n=46) | Wedge resection (n=128) |  |
| Age | 78.6 ± 3.3 | 78.0 ± 2.8 | 79.0 ± 3.5 | | 0.013 |
|  |  |  | 78.5 ± 3.5 | 79.1 ± 3.5 |  |
| Male | 111 (43.0) | 40 (47.6) | 71 (40.8) | | 0.300 |
|  |  |  | 21 (45.7) | 50 (39.1) |  |
| Body Mass Index | 24.2 ± 3.5 | 24.3 ± 2.9 | 24.2 ± 3.7 | | 0.873 |
|  |  |  | 24.4 ± 4.1 | 24.1 ± 3.6 |  |
| ECOG |  |  |  |  | 0.007 |
| 0, n (%) | 179 (69.4) | 49 (58.3) | 130 (74.7) | |  |
|  |  |  | 34 (73.9) | 96 (75.0) |  |
| ≥1, n (%) | 79 (30.6) | 35 (41.7) | 44 (25.3) | |  |
|  |  |  | 12 (26.1) | 32 (25.0) |  |
| Smoking status |  |  |  |  | 0.115 |
| Smoker (%) | 53 (20.5) | 18 (21.4) | 35 (20.1) | |  |
|  |  |  | 6 (13.1) | 29 (22.7) |  |
| Non-smoker (%) | 205 (79.5) | 66 (78.6) | 139 (80.0) | |  |
|  |  |  | 40 (87.0) | 99 (77.3) |  |
| Family history | 27 (10.5) | 11 (13.1) | 16 (9.2) | | 0.872 |
|  |  |  | 3 (6.5) | 13 (10.2) |  |
| Comorbidity (CCI) | 1.5 ± 1.4 | 1.5 ± 1.4 | 1.6 ± 1.4 | | 0.680 |
|  |  |  | 1.1 ± 1.2 | 1.7 ± 1.4 |  |
| PFT |  |  |  |  |  |
| FVC % | 108.6 ± 19.4 | 108.9 ± 18.5 | 108.4 ± 19.9 | | 0.141 |
|  |  |  | 102.7 ± 20.4 | 110.4 ± 19.5 |  |
| FEV1 % | 116.2 ± 27.7 | 114.4 ± 27.8 | 117.0 ± 27.7 | | 0.568 |
|  |  |  | 110.7 ± 31.8 | 119.1 ± 26.0 |  |
| Pre-operative CEA^a^ |  |  |  |  | 0.394 |
| ≧5 | 38 (14.7) | 10 (11.9) | 28 (16.1) | |  |
|  |  |  | 7 (15.2) | 21 (16.4) |  |
| < 5 | 216 (83.7) | 72 (85.7) | 144 (82.8) | |  |
|  |  |  | 38 (82.6) | 106 (82.8) |  |
| CT images |  |  |  |  |  |
| Total tumor diameter, cm |  |  |  |  | <0.001 |
| 0-1 | 27 (10.5) | 1 (1.2) | 26 (14.9) | |  |
|  |  |  | 2 (4.3) | 24 (18.8) |  |
| 1-2 | 92 (35.7) | 18 (21.4) | 75 (43.1) | |  |
|  |  |  | 13 (28.3) | 62 (48.4) |  |
| 2-3 | 85 (32.9) | 32 (38.1) | 53 (30.5) | |  |
|  |  |  | 22 (47.8) | 31 (24.2) |  |
| ≥3 | 54 (20.9) | 33 (39.3) | 20 (11.5) | |  |
|  |  |  | 9 (19.6) | 11 (8.6) |  |
| Solid component diameter, cm |  |  |  |  | <0.001 |
| 0-1 | 84 (32.6) | 8 (9.5) | 76 (43.7) | |  |
|  |  |  | 14 (30.4) | 62 (48.4) |  |
| 1-2 | 97 (37.6) | 32 (38.1) | 65 (37.4) | |  |
|  |  |  | 18 (39.1) | 47 (36.7) |  |
| 2-3 | 77 (29.8) | 44 (52.4) | 33 (19.0) | |  |
| C/T ratio (%) |  |  | 14 (30.4) | 19 (14.8) | 0.176 |
| 0-25% | 118 (45.7) | 33 (28.0) | 85 (48.9) | |  |
|  |  |  | 24 (52.2) | 61 (47.7) |  |
| 25-50% | 18 (7.0) | 3 (16.7) | 15 (8.6) | |  |
|  |  |  | 3 (6.5) | 12 (9.4) |  |
| ≥50% | 122 (47.3) | 48 (39.3) | 74 (42.5) | |  |
|  |  |  | 19 (41.3) | 55 (43.0) |  |
| Depth, cm | 0.9 ±0.8 | 1.0 ±0.9 | 0.8 ± 0.7 | | 0.007 |
|  |  |  | 0.9 ± 0.7 | 0.7 ± 0.7 |  |

Data are presented as mean ± SD or number (%).

^a^ 4 patients lacked the pre-operative CEA data.

Abbreviations: BMI, body mass index; CCI, Charlson Comorbidity Index; CEA, carcinoembryonic antigen; C/T ratio, consolidation-to-tumor ratio; ECOG, Eastern Cooperative Oncology Group performance status; FEV1, forced expiratory volume in 1 second; FVC, forced vital capacity; NSCLC, non-small-cell lung cancer; PFT, pulmonary function test.

| **Supplementary Table 2.** Pathological features before propensity score matching | | | | | | |  |
| --- | --- | --- | --- | --- | --- | --- | --- |
|  | Total  (n=258) | | Lobectomy  (n=84) | Sublobar resection  (n=174) | | P-value | |
|  |  |  |  | Segmentectomy (n=46) | Wedge resection (n=128) |  |  |
| Differentiation |  |  |  |  |  | 0.143 | |
| Well | 53 (20.5) | | 13 (15.5) | 40 (23.0) | |  | |
|  |  | |  | 8 (17.4) | 32 (25.0) |  | |
| Moderate and poor | 199 (77.1) | | 70 (83.3) | 129 (74.1) | |  | |
|  |  | |  | 38 (82.6) | 91 (71.1) |  | |
| VPI | 56 (21.7) | | 24 (28.6) | 33 (19.0) | | 0.063 | |
|  |  | |  | 11 (23.9) | 21 (16.4) |  | |
| LVI | 44 (17.1) | | 17 (20.2) | 27 (15.5) | | 0.217 | |
|  |  | |  | 7 (15.2) | 20 (15.6) |  | |
| Resection margin involvement | 13 (5.0) | | 2 (2.4) | 11 (6.3) | | 0.103 | |
|  |  | |  | 5 (10.9) | 6 (4.7) |  | |
| Resection margin (cm) | N/A | | N/A | 1.6±0.6 | | N/A | |
|  |  | |  | 1.1±0.7 | 1.7 ±0.6 |  | |
| STAS^a^ |  | |  |  |  | 0.577 | |
| positive | 37 (33.3) | | 8 (42.1) | 29(31.5) | |  | |
|  |  | |  | 7 (36.8) | 22 (30.1) |  | |
| negative | 74 (66.7) | | 11 (57.9) | 63(68.5) | |  | |
|  |  | |  | 12(63.2) | 51 (69.9) |  | |
| Pathological tumor diameter, cm |  | |  |  |  | <0.001 | |
| 0-1 | 23 (8.9) | | 2 (2.4) | 21 (12.1) | |  | |
|  |  | |  | 1 (2.2) | 20 (15.6) |  | |
| 1-2 | 86 (33.3) | | 14 (16.7) | 72 (41.4) | |  | |
|  |  | |  | 12 (26.1) | 60 (46.9) |  | |
| 2-3 | 87 (33.7) | | 30 (35.7) | 57 (32.8) | |  | |
|  |  | |  | 22 (47.8) | 35 (27.3) |  | |
| ≥3 | 62 (24.0) | | 38 (45.2) | 24 (13.8) | |  | |
|  |  | |  | 11 (23.9) | 13 (10.2) |  | |
| Histology |  | |  |  |  | 0.563 | |
| Adenocarcinoma | 227 (88.0) | | 71 (84.5) | 156 (89.7) | |  | |
|  |  | |  | 40 (87.0) | 116 (90.6) |  | |
| SqCC | 24 (9.3) | | 10 (11.9) | 14 (8.0) | |  | |
|  |  | |  | 6 (13.0) | 8 (6.2) |  | |
| Adenosquamous | 3 (1.2) | | 1 (1.2) | 2 (1.1) | |  | |
|  |  | |  | 0 (0.0) | 2 (1.6) |  | |
| Pleomorphic | 1 (0.4) | | 1 (1.2) | 0 (0.0) | |  | |
|  |  | |  | 0(0.0) | 0 (0.0)) |  | |
| Carcinoid | 2 (0.8) | | 1 (1.2) | 1 (0.6) | |  | |
|  |  | |  | 0(0.0) | 1 (0.8) |  | |
| Pathological TNM stage |  | |  |  |  | 0.636 | |
| IA | 204 (79.1) | | 51 (60.7) | 153 (87.9) | | <0.001 | |
| IA1 | 32 (12.4) | | 3 (3.6) | 29 (16.7) | |  | |
|  |  | |  | 1 (2.2) | 28 (21.9) |  | |
| IA2 | 84 (32.6) | | 15 (17.9) | 69 (39.7) | |  | |
|  |  | |  | 15 (32.6) | 54 (42.2) |  | |
| IA3 | 88 (34.1) | | 33 (39.3) | 55 (31.6) | |  | |
|  |  | |  | 20 (43.5) | 35 (27.3) |  | |
| IB | 54 (20.9) | | 33 (39.3) | 21 (12.1) | |  | |
|  |  | |  | 10 (21.7) | 11 (8.6) |  | |

Data are presented as mean ± SD or number (%).

^a^147 patients lacked the STAS data.

Abbreviations: LVI, lymphovascular invasion; NSCLC, non-small-cell lung carcinoma; STAS, Spread through air space; SqCC, squamous cell carcinoma; VPI, visceral pleural invasion.

| **Supplementary Table 3.** Perioperative outcomes before propensity score matching | | | | | |
| --- | --- | --- | --- | --- | --- |
|  | Total  (n=258) | Lobectomy (n=84) | Sublobar resection  (n=174) | | P-value |
|  |  |  | Segmentectomy (n=46) | Wedge resection (n=128) |  |
| VATS approach | 258 (100.0) | 84 (100.0) | 174 (100.0) | | >.99 |
|  |  |  | 46 (100.0) | 128 (100.0) |  |
| Operative time | 112.2 ±48.5 | 145.3 ±50.9 | 96.8 ±38.8 | | 0.425 |
|  |  |  | 126.6 ±45.3 | 85.0 ±26.1 |  |
| Operative bleeding | 15.3 ±50.9 | 32.5 ± 77.4 | 7.0 ±27.6 | | <0.001 |
|  |  |  | 16.3 ±38.1 | 2.7 ±22.1 |  |
| Dissected LNs |  |  |  |  |  |
| Total number | 8.0 ±7.1 | 12.4 ±7.8 | 5.8 ±5.7 | | <0.001 |
|  |  |  | 7.3 ±5.9 | 5.3 ±5.5 |  |
| Total station | 3.2 ±1.5 | 4.2 ±1.4 | 2.7 ±1.4 | | <0.001 |
|  |  |  | 3.4 ± 1.4 | 2.4 ±1.2 |  |
| Postoperative ICU stay | 0.6 ±1.2 | 0.7 ± 0.8 | 0.6 ±1.3 | | 0.365 |
|  |  |  | 0.6 ±0.6 | 0.6 ±1.5 |  |
| Post-op hospital stay | 5.6 ±5.1 | 7.5 ± 6.9 | 4.7 ±3.5 | | <0.001 |
|  |  |  | 5.0 ±2.1 | 4.5 ±3.9 |  |
| Chest tube days | 2.7 ±2.8 | 3.8 ± 4.1 | 2.1 ±1.7 | | <0.001 |
|  |  |  | 2.4 ±1.9 | 1.6 ±2.0 |  |
| Chest tube |  |  |  |  |  |
| Duration, days | 2.7 ±2.8 | 3.8 ± 4.1 | 2.1 ±1.7 | | <0.001 |
|  |  |  | 2.4 ±1.9 | 1.6 ±2.0 |  |
| Chest tube ≥3days | 88 (34.1) | 48 (57.1) | 40 (23.0) | | <0.001 |
|  |  |  | 12 (26.1) | 28 (21.9) |  |
| Chest tube >5days | 27 (10.5) | 15 (17.9) | 12 (6.9) | | 0.021 |
|  |  |  | 4 (8.7) | 8 (6.3) |  |
| Postoperative complications |  |  |  |  |  |
| All complications | 37 (14.3) | 22 (26.2) | 15 (8.6) | | <0.001 |
|  |  |  | 6 (13.0) | 9 (7.0) |  |
| Grade 3a or greater | 34 (13.2) | 19 (22.6) | 15 (8.6) | | 0.005 |
|  |  |  | 6 (13.0) | 9 (7.0) |  |
| Grade 3b or greater | 9 (3.5) | 4 (4.8) | 5 (2.9) | | 0.605 |
|  |  |  | 2 (4.3) | 3 (2.3) |  |
| Conversion to thoracotomy | 1 (0.4) | 1 (1.2) | 0 (0.0) | | >0.99 |
|  |  |  | 0 (0.0) | 0 (0.0) |  |
| 30-day mortality | 0 (0.0) | 0 (0.0) | 0 (0.0) | | >0.99 |
|  |  |  | 0 (0.0) | 0 (0.0) |  |

Data are presented as mean ± SD or number (%).

Abbreviations: ICU, intensive care unit; LNs, lymph nodes; NA, not available; TIA, transient ischemic attack; VATS, video-assisted thoracoscopic surgery.

**Supplementary Table 4a.** Details of postoperative complications, before matching

| Before matching | Total  (n=258) | Lobectomy  (n=84) | Sublobar resection  (n=174) | | P-value |
| --- | --- | --- | --- | --- | --- |
|  |  |  | Segmentectomy  (n=46) | wedge  (n=128) |  |
|  |  |  |  |  |  |
| **All complications** | 37 (14.3) | 22 (26.2) | 15 (8.6) | | <.001 |
|  |  |  | 6 (13.0) | 9 (7.0) |  |
| Grade 3a or greater | 34 (13.2) | 19 (22.6) | 15 (8.6) | | .002 |
|  |  |  | 6 (13.0) | 9 (7.0) |  |
| Grade 3b or greater | 9 (3.5) | 4 (4.8) | 5 (2.9) | | .44 |
|  |  |  | 2 (4.3) | 3 (2.3) |  |
| **Grade II** | 9 (3.5) | 6 (7.1) | 3 (1.7) | | .03 |
|  |  |  | 1 (2.2) | 2 (1.6) |  |
| Wound infection | 4 (1.6) | 1 (1.2) | 3 (1.7) | |  |
|  |  |  | 1 (2.2) | 2 (1.6) |  |
| GI occult bleeding needs transfusion | 1 (0.4) | 0 (0.0) | 1 (0.6) | |  |
|  |  |  | 0 (0.0) | 1 (0.8) |  |
| Hypoglycemia | 2 (0.8) | 2 (2.4) | 0 (0.0) | |  |
|  |  |  | 0 (0.0) | 0 (0.0) |  |
| Hematuria after foley removal | 1 (0.4) | 1 (1.2) | 0 (0.0) | |  |
|  |  |  | 0 (0.0) | 0 (0.0) |  |
| Urinary retention | 3 (1.2) | 3 (3.6) | 0 (0.0) | |  |
|  |  |  | 0 (0.0) | 0 (0.0) |  |
| Postoperative AKI | 1 (0.4) | 1 (1.2) | 0 (0.0) | |  |
|  |  |  | 0 (0.0) | 0 (0.0) |  |
| **Grade IIIa** | 31 (12.0) | 17 (20.2) | 14 (8.0) | | .01 |
|  |  |  | 6 (13.0) | 8 (6.3) |  |
| Pulmonary air leakage | 15 (5.8) | 6 (7.1) | 9 (5.2) | |  |
|  |  |  | 3 (6.5) | 6 (4.7) |  |
| Subcutaneous emphysema | 7 (2.7) | 4 (4.8) | 3 (1.7) | |  |
|  |  |  | 1 (2.2) | 2 (1.6) |  |
| Atelectasis | 7 (2.7) | 5 (6.0) | 2 (1.1) | |  |
|  |  |  | 1 (2.2) | 1 (0.8) |  |
| Pneumonia, empyema | 4 (1.6) | 2 (2.4) | 2 (1.1) | |  |
|  |  |  | 0 (0.0) | 2 (1.6) |  |
| Pleural effusion | 4 (1.6) | 2 (2.4) | 2 (1.1) | |  |
|  |  |  | 1 (2.2) | 1 (0.8) |  |
| Exacerbation of COPD | 2 (0.8) | 1 (1.2) | 1 (0.6) | |  |
|  |  |  | 1 (2.2) | 0 (0.0) |  |
| Delirium | 5 (1.9) | 3 (3.6) | 2 (1.1) | |  |
|  |  |  | 0 (0.0) | 2 (1.6) |  |
| Arrhythmia | 5 (1.9) | 3 (3.6) | 2 (1.1) | |  |
|  |  |  | 1 (2.2) | 1 (0.8) |  |
| Recurrent nerve paralysis | 2 (0.8) | 0 (0.0) | 2 (1.1) | |  |
|  |  |  | 1 (2.2) | 1 (0.8) |  |
| Cerebral vascular accident | 2 (0.8) | 1 (1.2) | 1 (0.6) | |  |
|  |  |  | 1 (2.2) | 0 (0.0) |  |
| **Grade IIIb** | 4 (1.6) | 3 (3.6) | 1 (0.6) | | .07 |
|  |  |  | 1 (2.2) | 0 (0.0) |  |
| Pulmonary air leakage treated by operation | 4 (1.6) | 3 (3.6) | 1 (0.6) | |  |
|  |  |  | 1 (2.2) | 0 (0.0) |  |
| **Grade IVa** | 4 (1.6) | 1 (1.2) | 3 (1.7) | | .75 |
|  |  |  | 1 (2.2) | 2 (1.6) |  |
| Empyema | 2 (0.8) | 1 (1.2) | 1 (0.6) | |  |
|  |  |  | 1 (2.2) | 0 (0.0) |  |
| Cardiac failure | 1 (0.4) | 1 (1.2) | 0 (0.0) | |  |
|  |  |  | 0 (0.0) | 1 (0.8) |  |
| Arrhythmia unstable vital sign | 2 (0.8) | 0 (0.0) | 2 (1.1) | |  |
|  |  |  | 0 (0.0) | 1 (0.8) |  |
| **Grade IVb** | 4 (1.6) | 2 (2.4) | 2 (1.1) | | .45 |
|  |  |  | 1 (2.2) | 1 (0.8) |  |
| Respiratory failure | 3 (1.2) | 2 (2.4) | 1 (0.6) | |  |
|  |  |  | 0 (0.0) | 1 (0.8) |  |
| Cardiac failure with dialysis | 1 (0.4) | 0 (0.0) | 1 (0.6) | |  |
|  |  |  | 1 (2.2) | 0 (0.0) |  |
| **Grade V** | 2 (0.8) | 2 (2.4) | 0 (0.0) | | .04 |
|  |  |  | 0 (0.0) | 0 (0.0) |  |
| Pneumonia to respiratory failure and death | 2 (0.8) | 2 (2.4) | 0 (0.0) | |  |
|  |  |  | 0 (0.0) | 0 (0.0) |  |
| **30-day mortality** | 0 (0.0) | 0 (0.0) | 0 (0.0) | |  |
|  |  |  | 0 (0.0) | 0 (0.0) |  |

Data are presented as number (%).

Abbreviations: AKI, acute kidney injury; COPD, chronic obstructive pulmonary disease; GI, gastrointestinal.

**Supplementary Table 4b.** Details of postoperative complications, After matching

| After matching | Total (n=120) | Lobectomy (n=60) | Sublobar resection  (n=60) | | P-value |
| --- | --- | --- | --- | --- | --- |
|  |  |  | Segmentectomy  (n=20) | Wedge  (n=40) |  |
| **All complications** | 23 (19.2) | 17 (28.3) | 6 (10.0) | | .01 |
|  |  |  | 2 (10.0) | 4 (10.0) |  |
| Grade 3a or greater | 21 (17.5) | 15 (25.0) | 6 (10.0) | | .03 |
|  |  |  | 2 (10.0) | 4 (10.0) |  |
| Grade 3b or greater | 4 (3.3) | 3 (5.0) | 1 (1.7) | | .31 |
|  |  |  | 0 (0.0) | 1 (2.5) |  |
| **Grade II** | 7 (5.8) | 5 (8.3) | 2 (3.3) | | .24 |
|  |  |  | 0 (0.0) | 0 (0.0) |  |
| Wound infection | 2 (1.7) | 1 (1.7) | 1 (1.7) | |  |
|  |  |  | 1 (5.0) | 0 (0.0) |  |
| GI occult bleeding needs transfusion | 1 (0.8) | 0 (0.0) | 1 (1.7) | |  |
|  |  |  | 0 (0.0) | 1 (2.5) |  |
| Hypoglycemia | 2 (1.7) | 2 (3.3) | 0 (0.0) | |  |
|  |  |  | 0 (0.0) | 0 (0.0) |  |
| Hematuria after foley removal | 1 (0.8) | 1 (1.7) | 0 (0.0) | |  |
|  |  |  | 0 (0.0) | 0 (0.0) |  |
| Urinary retention | 2 (1.7) | 2 (3.3) | 0 (0.0) | |  |
|  |  |  | 0 (0.0) | 0 (0.0) |  |
| Postoperative AKI | 1 (0.8) | 1 (1.7) | 0 (0.0) | |  |
|  |  |  | 0 (0.0) | 0 (0.0) |  |
| **Grade IIIa** | 20 (16.7) | 14 (23.3) | 6 (10.0) | | .05 |
|  |  |  | 2 (10.0) | 4 (5.0) |  |
| Pulmonary air leakage | 9 (7.5) | 7 (11.7) | 2 (3.3) | |  |
|  |  |  | 0 (0.0) | 2 (2.5) |  |
| Subcutaneous emphysema | 6 (5.0) | 4 (6.7) | 2 (3.3) | |  |
|  |  |  | 0 (0.0) | 2 (2.5) |  |
| Atelectasis | 5 (4.2) | 5 (8.3) | 0 (0.0) | |  |
|  |  |  | 0 (0.0) | 0 (0.0) |  |
| Pneumonia, empyema | 1 (0.8) | 1 (1.7) | 0 (0.0) | |  |
|  |  |  | 0 (0.0) | 0 (0.0) |  |
| Pleural effusion | 3 (2.5) | 2 (3.3) | 1 (1.7) | |  |
|  |  |  | 0 (0.0) | 1 (2.5) |  |
| Exacerbation of COPD | 2 (1.7) | 2 (3.3) | 0 (0.0) | |  |
|  |  |  | 0 (0.0) | 0 (0.0) |  |
| Delirium | 5 (4.2) | 3 (5.0) | 2 (3.3) | |  |
|  |  |  | 0 (0.0) | 2 (5.0) |  |
| Arrhythmia | 3 (2.5) | 2 (3.3) | 1 (1.7) | |  |
|  |  |  | 0 (0.0) | 1 (2.5) |  |
| Recurrent nerve paralysis | 1 (0.8) | 0 (0.0) | 1 (1.7) | |  |
|  |  |  | 1 (5.0) | 0 (0.0) |  |
| Cerebral vascular accident | 1 (0.8) | 0 (0.0) | 1 (1.7) | |  |
|  |  |  | 1 (5.0) | 0 (0.0) |  |
| **Grade IIIb** | 2 (1.7) | 2 (3.3) | 0 (0.0) | | .15 |
|  |  |  | 0 (0.0) | 0 (0.0) |  |
| Pulmonary air leakage treated by operation | 2 (1.7) | 2 (3.3) | 0 (0.0) | |  |
|  |  |  | 0 (0.0) | 0 (0.0) |  |
| **Grade IVa** | 1 (0.8) | 1 (1.7) | 0 (0.0) | | .32 |
|  |  |  | 0 (0.0) | 0 (0.0) |  |
| Empyema | 1 (0.8) | 1 (1.7) | 0 (0.0) | |  |
|  |  |  | 0 (0.0) | 0 (0.0) |  |
| Cardiac failure | 0 (0.0) | 0 (0.0) | 0 (0.0) | |  |
|  |  |  | 0 (0.0) | 0 (0.0) |  |
| Arrhythmia unstable vital sign | 0 (0.0) | 0 (0.0) | 0 (0.0) | |  |
|  |  |  | 0 (0.0) | 0 (0.0) |  |
| **Grade IVb** | 3 (2.5) | 2 (3.3) | 1 (1.7) | | .56 |
|  |  |  | 0 (0.0) | 1 (2.5) |  |
| Respiratory failure | 3 (2.5) | 2 (3.3) | 1(1.7) | |  |
|  |  |  | 0 (0.0) | 1 (2.5) |  |
| Cardiac failure with dialysis | 1 (0.8) | 0 (0.0) | 1 (1.7) | |  |
|  |  |  | 0 (0.0) | 1 (2.5) |  |
| **Grade V** | 2 (1.7) | 2 (3.3) | 0 (0.0) | | .15 |
|  |  |  | 0 (0.0) | 0 (0.0) |  |
| Pneumonia to respiratory failure and death | 2 (1.7) | 2 (3.3) | 0 (0.0) | |  |
|  |  |  | 0 (0.0) | 0 (0.0) |  |
| **30-day mortality** | 0 (0.0) | 0 (0.0) | 0 (0.0) | |  |
|  |  |  | 0 (0.0) | 0 (0.0) |  |

Data are presented as number (%).

Abbreviations: AKI, acute kidney injury; COPD, chronic obstructive pulmonary disease; GI, gastrointestinal.

**Supplementary Table 5.** Recurrence patterns of pStage I non-small cell lung cancer patients who have undergone surgery

|  | Total  (n=258) | Lobectomy  (n=84) | Sublobar  (n=174) | | *p* value |
| --- | --- | --- | --- | --- | --- |
|  |  |  | Segmentectomy  (n=46) | Wedge  (n=128) |  |
| Total recurrence number | 33 (12.8) | 14 (16.7) | 19 (10.9) | | .195 |
|  |  |  | 5 (10.9) | 14 (10.9) |  |
| Local | 11 (4.3) | 3 (3.6) | 8 (4.6) | | .702 |
|  |  |  | 2 (4.3) | 6 (4.7) |  |
| Intersegmental line | 7 (2.7) | 2 (2.4) | 5 (2.9) | |  |
|  |  |  | 1 (2.2) | 4 (3.1) |  |
| Bronchial stump | 4 (1.6) | 1 (1.2) | 3 (1.7) | |  |
|  |  |  | 1 (2.2) | 2 (1.6) |  |
| Regional | 8 (3.1) | 5 (6.0) | 3 (1.7) | | .066 |
|  |  |  | 1 (2.2) | 3 (2.3) |  |
| Ipsilateral lung | 3 (1.2) | 2 (2.4) | 1 (0.6) | |  |
|  |  |  | 0 (0.0) | 1 (0.8) |  |
| Intrathoracic lymph node | 2 (0.8) | 1 (1.2) | 1 (0.6) | |  |
|  |  |  | 1 (2.2) | 0 (0.0) |  |
| Ipsilateral pleural seeding/ ipsilateral malignant effusion | 3 (1.2) | 2 (2.4) | 1 (0.6) | |  |
|  |  |  | 0 (0.0) | 1 (0.8) |  |
| Distal | 14 (5.4) | 6 (7.1) | 8 (4.6) | | .398 |
|  |  |  | 3 (6.5) | 5 (3.9) |  |
| Contralateral lung | 8 (3.1) | 4 (4.8) | 4 (2.2) | |  |
|  |  |  | 1 (2.2) | 2 (1.6) |  |
| Bone | 5 (1.9) | 2 (2.4) | 3 (1.7) | |  |
|  |  |  | 2 (4.3) | 1 (0.8) |  |
| Brain | 3 (1.2) | 2 (2.4) | 1 (0.6) | |  |
|  |  |  | 1 (2.2) | 0 (0.0) |  |
| Liver | 2 (0.8) | 0 (0.0) | 2 (1.1) | |  |
|  |  |  | 0 (0.0) | 2 (1.6) |  |
| Others | 0 (0.0) | 0(0.0) | 0 (0.0) | |  |
|  |  |  | 0 (0.0) | 0 (0.0) |  |

Data are presented as number (%).

**Supplementary Table 6.** Mortality patterns of geriatric pStage I NSCLC patients undergoing lobectomy or sublobar resection

|  | | Total  (n=258) | Lobectomy(n=84) | Sublobar resection  (n=174) | |  |  |
| --- | --- | --- | --- | --- | --- | --- | --- |
|  | |  |  | Segmentectomy (n=46) | Wedge  (n=128) | P-value |  |
| Overall mortality | | 19 (7.4) | 8 (9.5) | 11 (6.3) | | .36 |  |
|  | |  |  | 3 (6.5) | 8 (6.3) |  |  |
| Lung cancer-specific death | | 3 (1.2) | 0 (0.0) | 3(1.7) | | .23 |  |
| (Metastasis related organ failure) | |  |  | 0 (0.0) | 3 (2.3) |  |  |
|  | |  |  |  |  |  |  |
| Non-lung cancer-specific death | | 16 (6.2) | 8 (9.5) | 8 (4.6) | | .12 |  |
|  | |  |  | 3 (6.5) | 5 (3.9) |  |  |
| Pneumonia | | 11 (4.3) | 7 (8.3) | 4 (2.3) | |  |  |
|  | |  |  | 1 (2.2) | 3 (2.3) |  |  |
| GI bleeding | | 1 (0.4) | 1 (1.2) | 0 (0.0) | |  |  |
|  | |  |  | 0 (0.0) | 0 (0.0) |  |  |
| Ileus related IAI | | 1 (0.4) | 0 (0.0) | 1 (0.6) | |  |  |
|  | |  |  | 1 (2.2) | 0 (0.0) |  |  |
| Hemodialysis catheter infection | | 1 (0.4) | 0 (0.0) | 1 (0.6) | |  |  |
|  | |  |  | 0 (0.0) | 1 (0.8) |  |  |
| Other malignancy-related death^a^ | | 2 (0.8) | 0 (0.0) | 2 (1.2) | |  |  |
|  | |  |  | 1 (2.2) | 1(0.8) |  |  |
|  | Data are presented as number (%).  ^a^Two patients died due to pancreatic cancer metastasis and colon cancer metastasis. | | | | | | |

**Supplementary Table 7.** Univariable analyses of correlations between clinicopathological features and lung cancer-related overall survival

| Variables | Hazard ratio | 95% Confidence interval | P-value |
| --- | --- | --- | --- |
| Age(years) |  |  |  |
| ≥80 vs < 80years | 4.824 | 0.434-53.586 | .20 |
| Sex |  |  |  |
| Female vs Male | 1.590 | 0.144-17.567 | .71 |
| ECOG |  |  |  |
| ≥1 vs 0 | 0.883 | 0.079-9.846 | .92 |
| Smoking | 7.646 | 0.692-84.421 | .10 |
| Lung cancer family history | 4.061 | 0.368-44.816 | .25 |
| Charlson comorbidity index |  |  |  |
| ≥2 vs <2 | 1.532 | 0.139-16.907 | .73 |
| CEA level (ng/mL) |  |  |  |
| ≥5 vs <5 | 0.040 | 0-131312.735 | .68 |
| Pulmonary function test |  |  |  |
| FEV1 <80% vs ≥80% | 0.045 | 0-37573361.22 | .77 |
| FEV1/FVC <75% vs ≥75% | 3.926 | 0.353-43.686 | .27 |
| C/T ratio |  |  |  |
| ≥50% vs <50% | 0.473 | 0.043-5.238 | .54 |
| VPI | 1.878 | 0.170-20.741 | .61 |
| LVI | 2.533 | 0.228-28.094 | .45 |
| Surgical method |  |  |  |
| Sublobar vs Lobectomy | 79.331 | 0.005-1159772.306 | .37 |

Abbreviations: CCI, Charlson Comorbidity Index; CEA, carcinoembryonic antigen; CT image, computed tomography image; C/T ratio, consolidation-to-tumor ratio; ECOG, Eastern Cooperative Oncology Group performance status; FEV1, forced expiratory volume in 1 second; FVC, forced vital capacity; LVI, lymphovascular invasion; VPI, visceral pleural invasion.

**Supplementary Table 8.** Univariable analyses of correlations between clinicopathological features and non-lung cancer-related overall survival

| Variables | Hazard ratio | 95% Confidence interval | P-value |
| --- | --- | --- | --- |
| Age(years) |  |  |  |
| ≥80 vs < 80 years | 2.267 | 0.849-6.050 | .10 |
| Sex |  |  |  |
| Female vs Male | 0.249 | 0.080-0.773 | .02 |
| ECOG |  |  |  |
| ≥1 vs 0 | 2.097 | 0.776-5.663 | .14 |
| Smoking | 0.399 | 0.145-1.101 | .08 |
| Lung cancer family history | 1.056 | 0.240-4.647 | .94 |
| Charlson comorbidity index |  |  |  |
| ≥2 vs <2 | 1.722 | 0.628-4.744 | .29 |
| CEA level (ng/mL) |  |  |  |
| ≥5 vs <5 | 0.454 | 0.128-1.615 | .22 |
| Pulmonary function test |  |  |  |
| FEV1 <80% vs ≥80% | 0.045 | 0.0001-634.773 | .53 |
| FEV1/FVC <75% vs ≥75% | 1.262 | 0.449-3.548 | .66 |
| CT total diameter |  |  |  |
| 1-2 cm vs 0-1 cm | 0.806 | 0.089-7.288 | .85 |
| 2-3 cm vs 0-1 cm | 1.044 | 0.124-8.805 | .97 |
| ≥3 cm vs 0-1 cm | 1.630 | 0.189-14.070 | .66 |
| CT solid diameter |  |  |  |
| 1-2 cm vs 0-1 cm | 1.556 | 0.388-6.233 | .53 |
| 2-3 cm vs 0-1 cm | 2.052 | 0.529-7.963 | .30 |
| C/T ratio |  |  |  |
| ≥50% vs <50% | 2.248 | 0.779-6.488 | .13 |
| Depth |  |  |  |
| ≥1 cm vs <1cm | 1.488 | 0.556-3.981 | .43 |
| Pathological tumor diameter |  |  |  |
| 1-2 cm vs 0-1 cm | 0.260 | 0.016-4.167 | .34 |
| 2-3 cm vs 0-1 cm | 1.766 | 0.220-14.195 | .59 |
| ≥3 cm vs 0-1 cm | 1.638 | 0.196-13.702 | .65 |
| VPI | 1.733 | 0.602-4.992 | .31 |
| LVI | 3.025 | 1.099-8.329 | .03 |
| Surgical method |  |  |  |
| Sublobar vs Lobectomy | 0.910 | 0.332-2.492 | .85 |

Abbreviations: CEA, carcinoembryonic antigen; CT image, computed tomography image; C/T ratio, consolidation-to-tumor ratio; ECOG, Eastern Cooperative Oncology Group performance status; FEV1, forced expiratory volume in 1 second; FVC, forced vital capacity; LVI, lymphovascular invasion; VPI, visceral pleural invasion.

|  | Non-cancer-related overall survival | | |
| --- | --- | --- | --- |
| Variables | Hazard ratio | 95% Confidence interval | P-value |
| Sex |  |  |  |
| Female vs. Male | 0.271 | 0.078-0.936 | .049 |
| Smoker | 1.185 | 0.0.384-3.655 | .77 |
| LVI | 2.681 | 0.933-7.700 | .07 |
| Surgical method |  |  |  |
| Sublobar resection vs. Lobetomy | 1.106 | 0.309-3.138 | .85 |

**Supplementary Table 9.** Multivariable analyses of correlations between clinicopathological features and non-lung cancer-related overall survival

Abbreviations: LVI, lymphovascular invasion.
